# Evaluation of the Psychometric Properties of the Arab Compulsive Internet Use Scale (CIUS) by Item Response Theory Modeling (IRT)

**DOI:** 10.3390/ijerph191912099

**Published:** 2022-09-24

**Authors:** Yasser Khazaal, Fares Zine El Abiddine, Louise Penzenstadler, Djamal Berbiche, Ghada Bteich, Saeideh Valizadeh-Haghi, Lucien Rochat, Sophia Achab, Riaz Khan, Anne Chatton

**Affiliations:** 1 Addiction Medicine, Department of Psychiatry, Lausanne University Hospital and Lausanne University, 1015 Lausanne, Switzerland; 2 Department of Psychiatry and Addictology, Montréal University, Montréal, QC H3T 1J4, Canada; 3 Laboratory Psychological and Educational Research, Department of Psychology, University Djillali Liabes of Sidi Bel Abbes, Sidi Bel Abbes 22000, Algeria; 4 Addiction Unit, Department of Psychiatry, Geneva University Hospitals, 1205 Geneva, Switzerland; 5 Charles-LeMoyne Hospital Research Centre, Sherbrooke University, Sherbrooke, QC J1K 2R1, Canada; 6 Faculty of Public Health, Lebanon University, Tripoli P.O. Box 6573/14, Lebanon; 7 Department of Medical Library and Information Sciences, School of Allied Medical Sciences, Shahid Beheshti University of Medical Sciences, Tehran, Iran; 8 Department of Mental Health and Psychiatry, Frontier Medical College Affiliated to Bahria University Islamabad, Abbottabad 22010, Pakistan; 9 Department of Psychiatry, Geneva University Hospitals, 1205 Geneva, Switzerland

**Keywords:** Compulsive Internet Use Scale, compulsive internet use, internet addiction, item response theory

## Abstract

Introduction: The psychometric properties of the Arab translation of the Compulsive Internet Use Scale (CIUS) have been previously studied by confirmatory factor analysis (CFA) with AMOS software using the asymptotically distribution-free (ADF) estimator. Unidimensionality has been achieved at the cost of correlating several item variance errors. However, several reviews of SEM software packages and estimation methods indicate that the option of robust standard errors is not present in the AMOS package and that ADF estimation may yield biased parameter estimates. We therefore explored a second analysis through item response theory (IRT) using the parametric graded response model (GRM) and the marginal maximum likelihood (MML) estimation method embedded in the LTM package of R software. Differential item functioning (DIF) or item bias across subpopulations was also explored within IRT framework as different samples were investigated. The objective of the current study is to (1) analyze the Arab CIUS scale with IRT, (2) investigate DIF in three samples, and (3) contribute to the ongoing debate on Internet-use-related addictive behaviors using the CIUS items as a proxy. Methods: We assessed three samples of people, one in Algeria and two in Lebanon, with a total of 1520 participants. Results: Almost three out of every five items were highly related to the latent construct. However, the unidimensionality hypothesis was not supported. Furthermore, besides being locally dependent, the scale may be weakened by DIF across geographic regions. Some of the CIUS items related to increasing priority, impaired control, continued use despite harm, and functional impairment as well as withdrawal and coping showed good discriminative capabilities. Those items were endorsed more frequently than other CIUS items in people with higher levels of addictive Internet use. Conclusions: Contrary to earlier ADF estimation findings, unidimensionality of the CIUS scale was not supported by IRT parametric GRM in a large sample of Arab speaking participants. The results may be helpful for scale revision. By proxy, the study contributes to testing the validity of addiction criteria applied to Internet use related-addictive behaviors.

## 1. Introduction

In spite of many benefits associated with Internet use [1] including health-related ones via information [2,3], peer support [4], digital interventions [5,6,7,8,9,10], or remote teaching during the COVID-19 pandemic [11,12], a part of the population experiences excessive use of some Internet-delivered services [13,14], with increasing concerns about the young adults and adolescents [15,16]. This phenomenon is described as a compulsive and uncontrolled Internet use that leads to social or work-related functional impairment and distress [17,18]. It includes both excessive gaming [19,20] and non-gaming internet activities [21] such as social-network use [22], dating apps [23], smartphone use [24,25,26,27], cybersex and porn use [28,29,30], as well as Internet gambling [31] or compulsive health-information seeking [32]. Compulsive Internet use is often associated with comorbid psychopathologies, including depression, anxiety, attention deficit hyperactivity disorder, and obsessive–compulsive disorder [33,34,35,36]. Internet is a vehicle for many different behaviors such as gaming, gambling, porn, social network, and so on. Such behaviors differ from each other in several ways including variations in motives for use and specific rewards [23,37]. For such reasons, compulsive internet use was considered as an umbrella term for possible different behavioral addictions [38].

Accordingly, the fifth revision of the Diagnostic and Statistical Manual of Mental Disorders (DSM-5) focused more specifically on Internet gaming [39] rather than on other Internet addiction [21]. In its section III (conditions requiring further research), the so-called “Internet gaming disorder (IGD)” [40] was introduced. This is defined as a “persistent and recurrent use of the Internet to engage in games…leading to significant impairment or distress during the past 12 months as indicated by 5 or more out of 9 criteria” (i.e., preoccupation, withdrawal, tolerance, unsuccessful attempts to stop/limit, loss of interests due to gaming, continued use despite harm, deception, escape, and harm) [41].

Based on the evidence of a number of neurobiological, phenomenological, developmental, and functional impairment similarities between gaming disorder and other addictive disorders [42,43], the suggested diagnostic criteria are derived from substance use disorder and gambling disorder. The adequacy of such adaptation was criticized in numerous comments [44,45] due to possible confirmation biases in such an approach [46], which may lead to increased risk of misdiagnosis [47]. It was, for example, argued that employing symptoms of substance use disorder to gaming pathologizes behaviors that may be unproblematic in common video gamers too frequently [47]. For instance, symptoms such as withdrawal and tolerance [45,48,49], “preoccupation” [50,51], “escape” [50,52], and “deception” [52] have been found to poorly discriminate between healthy and “problematic” patterns of gaming.

Following DSM-5 efforts to propose diagnostic criteria for addictive use of gaming, the World Health Organization developed a proposal for Gaming Disorder in the eleventh revision of the International Classification of Diseases ICD-11 [46,53]. The guidelines for ICD-11 Gaming Disorder are impaired control, increasing priority given to gaming, and continued use despite harm and functional impairment whereas criteria such as withdrawal, tolerance, and escape are not included [54].

Different tools have been developed worldwide to assess addictive Internet use [50,55,56]. One of the most used is the Compulsive Internet Use Scale (CIUS) that provides a severity assessment of general problematic Internet use [57]. The 14 items of the CIUS covers the following main components of addictive behaviors: loss of control (items 1, 2, 5, and 9), preoccupation (items 4, 6, and 7); withdrawal symptoms (item 14); coping or mood modification (items 12 and 13); and conflict (items 3, 8, 10, and 11). Therefore, in the context of debates related to addictive gaming and Internet use, the CIUS could be used to assess the extent to which each criterion contributes to the severity of the disorder.

The CIUS, however, did not focus on gaming but rather assesses general Internet use. The instrument was successfully adapted to assess specific Internet use behavior (i.e., by asking people to answer the questions for the specific behavior) [28,32,58]. In spite of the specificities of each given Internet use behavior, several conditions may fit the category of “other specified disorders due to addictive behaviors” [21].

In 2022, about 465 million people live in Arab-speaking countries [59]. Internet access growth was particularly important in Arab-speaking countries in the last decade because of the increased penetration of mobile 3G networks [60]. Unfortunately, despite the growing importance of Internet use in these countries, there is still a lack of studies related to problematic Internet use among Arab-speaking people [61,62,63,64].

The psychometric properties of the CIUS have been previously studied by confirmatory factor analysis (CFA) with AMOS software using an asymptotically distribution-free (ADF) estimation procedure. However, several reviews of SEM software packages and estimation methods show that the option of robust standard errors is not present in AMOS package [65] and that ADF estimation may exhibit biased parameter estimates when the sample size is small [66] or when the number of indicators is large relative to the sample size [67]. Precision measures such as robust standard errors are important to assess how close an estimate is to a population value. A second analysis is thus performed by item response theory (IRT), a well-established, modern modeling paradigm used for measuring psychological constructs and their items. IRT offers a number of estimation methods [68], one of which is the marginal maximum likelihood (MML) method used in the LTM package of R software [69].

IRT is a family of models that uses latent characterizations of individuals and items as predictors of observed responses [70]. More interestingly, within the IRT family, the logistic graded-response model (GRM), one of the two-parameter logistic (2-PL) models, developed by Samejima, is specifically designed for the analysis of polytomous ordered categorical items [71]. With GRM, the severity of the characteristic of interest, here the addiction severity, can be estimated and severity grades can be assigned to each respondent [72].

In its traditional form, IRT modeling assumes three assumptions: unidimensionality, local independence, and monotonicity [73]. The unidimensionality assumption stipulates that the items of a test are a function of only one continuous latent construct. This assumption is analogous to that of the homogeneity assumption in analysis of analysis of variance [70]. The second assumption, local independence, states that the responses to an item are independently conditional on the person’s location on the latent continuum [74]. Monotonicity assumption refers to the functional form of the logistic curves resulting from the GRM model. It supposes that as the latent trait increases, the probability to endorse a higher item response category increases.

To the best of our knowledge, no study has tested the psychometric properties of the CIUS with IRT. The first objective of this study is thus to explore the psychometric properties of the CIUS using IRT modeling.

When assessing the existence of a common factor model across populations, the assumption of invariance, that is, whether the items used have the same meaning to respondents across groups, must be verified [75]. If this property cannot be established, it is difficult to determine if the differences observed are due to true differences or to different psychometric responses to the items. When the latent trait is associated with group differences, or in other words when invariance does not hold, this phenomenon is called Differential Item Functioning (DIF) [76]. Such items must be carefully examined as they may compromise the validity of the test.

Arabic countries are not a homogeneous entity. Cultural contrasts may exist among them. Thus, a second aim of this study was to investigate a possible DIF presence in this population within IRT framework.

In the context of the debates related to IGD criteria, a further aim of this study was to contribute to the discussion using the data driven by the analyses on Arab-speaking samples.

## 2. Methods

### 2.1. Participants and Procedure

This study combines three different samples totalizing 1520 participants. As 205 of them completed only demographics, the final sample size was *n* = 1315. One sample was from Algeria involving 592 participants and two from previous studies carried out in Lebanon involving 928 persons [61,77]. All participants gave written informed consent, and the questionnaires were completed anonymously. They were mainly women (62.4%), young (88.1%, aged between 15 and 30) and had a mean CIUS of 34.5 (standard deviation: SD = 10.9). Table 1 presents these characteristics.

No compensation was given. The sample from Algeria comprised students from the Djillali Liabes of Sidi Bel Abbes University recruited during regular classes. Data were collected using the paper/pencil method.

### 2.2. Instrument: Compulsive Internet Use Scale (CIUS)

The 14 original items of the CIUS were translated into Arabic [57] using a translation and back-translation method [61]. All items are scored on a Likert scale (1 = never, 2 = rarely, 3 = sometimes, 4 = often, and 5 = very often), with higher scores indicating higher levels of Internet addiction. The distribution of item responses at the country level can be viewed in Table 2. The scale has a good homogeneity yielding a Cronbach value of 0.87.

Up to now, only one study [60], to our knowledge, has examined this specific issue through a validation process. The CIUS has been shown to be unidimensional after allowing several item variance errors to be correlated [61].

### 2.3. Statistical Analysis

GRM is designed for the analysis of ordered polytomous variables [71]. This particularity makes it suitable for the analysis of the CIUS scale with its 14 survey questions measuring Internet addiction. The items are ranked on a 5-point Likert scale from 1 (*never*) to 5 (*very often*). Discrimination and threshold parameters are the two main estimates in GRM. As the latter is basically an ordered logistic model, the threshold parameters of each item are naturally estimated in increasing order and the number of threshold estimates is equal to the number of item categories minus 1. As each CIUS item has 5 categories, four thresholds were estimated for each item. Hence, the probability that a person’s response falls at or above a particular category given the latent trait is expressed as follows:

$$Pr\left(Y_{ij}\geq k|\theta_{j}\right)=\frac{exp\left\{a_{i}\left(\theta_{j}-b_{ik}\right)\right\}}{1+exp\left\{a_{i}\left(\theta_{j}-b_{ik}\right)\right\}}\theta_{j}\sim N\left(0,1\right)$$

where:

- $a_{i}$ represents the discrimination of item i,
- $b_{ik}$ is the kth cutpoint for item i,
- and $\theta_{j}$ is the latent trait of person *j*.

The discrimination parameter (or slope) refers to the differential capability of an item. It also reflects the strength of association between an item and the construct being measured. A high discrimination parameter value means that the probability of endorsing an item response increases more rapidly as the latent trait or severity increases [78]. The value of the slope parameter also quantifies the amount of information of an item. When this value is high, most of the information is concentrated along a small part of the latent trait range. In reverse, the information contained in items with low discrimination is scattered along a greater part of this range.

Some descriptive rules of thumb allow for a better interpretation of the discrimination parameter value as follows: 0 = non-discriminative power; 0.01–0.34 = very low; 0.35–0.64 = low; 0.65–1.34 = moderate; 1.35–1.69 = high; >1.70 = very high; + infinity = perfect [72].

As for the threshold parameters, they reflect the point along the latent continuum where an individual has a 50% chance of endorsing a particular question [79].

Both latent trait scores and thresholds are on the same z-score metric with mean 0 and unit standard deviation [70].

GRM is derived in terms of cumulative probabilities, and the resulting plots are called Item Characteristic Curves (ICC). The latter are graphical functions that represent the respondent’s latent trait as a function of the probability of endorsing an item [80]. We present these ICCs along with Item Information Curves (IICs), which tell us how much information each ICC provides. The shape of an IIC is determined both by its discrimination and by its threshold parameters, but the steepness of the curves is determined by the magnitude of the discrimination index. Each item contribution can be summed in turn to obtain the total scale information function (TIF), which tells us how accurately the tool can appraise person location estimates. The plot show the amount of psychometric information at each point along a latent severity dimension [81].

### 2.4. Model Assumptions and Fit

#### 2.4.1. Unidimensionality

Unidimensionality was evaluated using two different approaches: the goodness-of-fit of the model through the Root Mean Square Error of Approximation (RMSEA), the Comparative Fit Index (CFI), and the Standardized Root Mean Square Residual. (SRMR). Acceptable fits are indicated by RMSEA < 0.08, CFI values > 0.90, and SRMR < 0.08 [82,83]. The other approach used the Loevinger’s H coefficients, a non-parametric method for assessing the strength of the dimensionality of a scale. The scale is weakly unidimensional if 0.3 ≤ H < 0.4, moderate if 0.4 ≤ H < 0.5), and strong if H > 0.5 [84,85]. We used the Mokken package (72) of the R program [86] for that purpose.

#### 2.4.2. Local Independence

This assumption was tested through the item residual correlation matrix after fitting the model. Residual pairs > 0.1 are an indication for local dependence [87,88].

#### 2.4.3. Monotonicity

This assumption was examined through the rest-score graphs as the difference between the raw scale score and the item score for each item. These graphs picture the rest-scores on the *x*-axis and the proportion of respondents in each rest-score group endorsing the item on the *y*-axis. The Mokken package [89] was used to plot these graphs.

The above assumptions being addressed, the next step was to investigate potential DIF effects across geographic regions using an iterative hybrid ordinal logistic regression and Monte Carlo simulations implemented in the R Lordif package [90].

#### 2.4.4. Missing Values

Among the 1520 participants recruited, 1315 completed the CIUS questionnaire while the other 205 (13.5%) only completed demographics. Hence, they were not included in the analyses. 

#### 2.4.5. Sample Size Requirement

There are no formal answers in the literature regarding sample size requirements, but some guidelines are offered either by simulation studies [91] or through rules of thumb [91,92]. They suggest sample sizes ranging from 250 to 500 for satisfactory IRT analyses. The sample size considered in this study was 1315 participants.

#### 2.4.6. Statistical Software

We took advantage of the free R program [86], one of the most powerful statistical software, to conduct the analyses. It provides all the necessary packages, one of which is the widely used LTM, to analyze IRT-GRM models.

## 3. Results

### 3.1. Unidimensionality

The goodness-of-fit statistics of the model were satisfactory with RMSEA = 0.065, CFI = 0.902 and SRMR = 0.069. However, the Loevinger’s H coefficient yielded a value of 0.33, indicating that the scale is weakly unidimensional.

### 3.2. Local Independence

A number of item pairs showed problematic covariation and for which residual correlations as high as 0.25 were highlighted, a value which far exceeded the 0.10 cut-off limit. These findings strongly suggest that the scale is not exempted from local dependency bias.

### 3.3. Monotonicity

The monotonicity assumption was satisfied as the probability of endorsing higher response categories increased as the latent trait increases.

#### 3.3.1. IRT Graded Parameter Estimates

**Figure 1:**
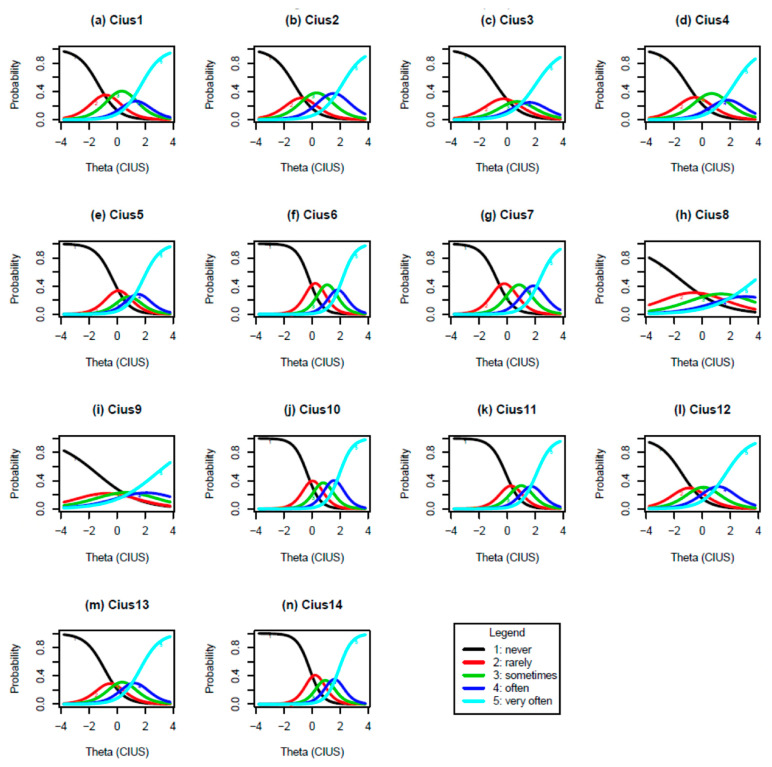
Item characteristic curves.

**Figure 2:**
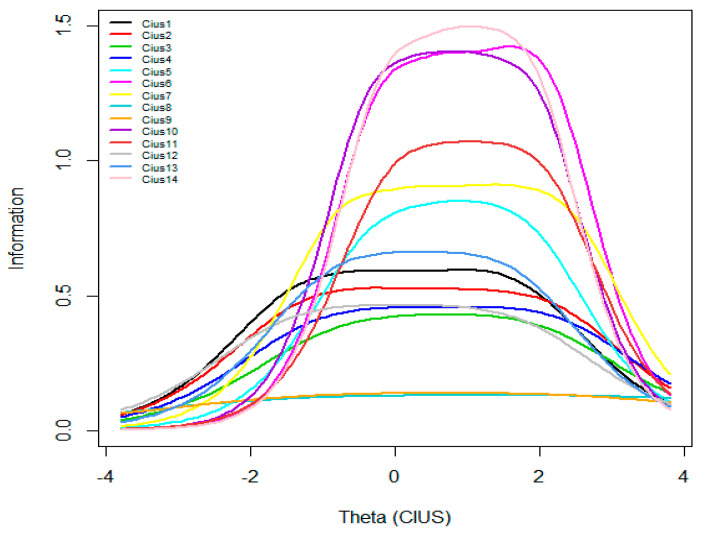
Item information curves.

**Figure 3:**
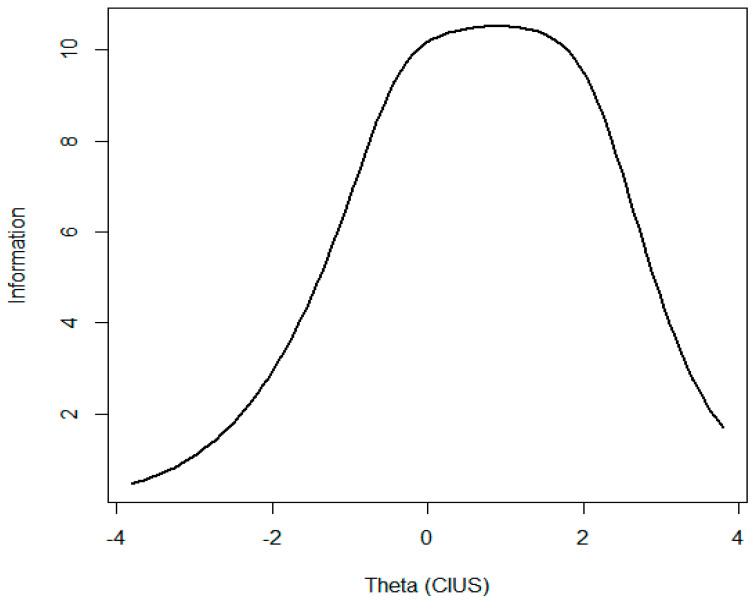
Total (scale) information curve.

GRM parameter estimates are reported in Table 3. Figure 1, Figure 2 and Figure 3 present ICC, IIC, and TIF curves.

From Table 3, it can be seen that, in terms of the ranges suggested by Baker, Item 8 had low discriminative power, Items 2, 3, 4, 9, and 12 had moderate discriminative power, Items 1, 5, and 13 had high discriminative power, and Items 6, 7, 10, 11, and 14 had very high discriminative power. Thus, the discriminative power of all items ranged from 0.64 to 2.20. Besides providing reasonably good differentiation among individuals, large values of discrimination parameter estimates also indicate that the items concerned are highly related to the latent variable, Internet addiction. Table 3 also shows that all threshold estimates go in an increasing order from negative to positive values. This means that they span a broad range of the latent trait below and above the mean. However, item 6 whose thresholds are −0.21, 0.67, 1.49, and 2.16 seems to be better at differentiating people above the mean. In terms of cumulative comparisons, a person with θ =−0.21 has a 50% chance of answering 1 versus greater than or equal to 2, a person with θ = 0.67 has a 50% chance of answering 1 or 2 versus greater than or equal to 3, a person with θ = 1.49 has a 50% chance of answering 1 or 2 or 3 versus greater than or equal to 4, and a person with θ = 2.16 has a 50% chance of answering 1 or 2 or 3 or 4 versus 5.

Figure 1 displays 14 ICCs. These curves illustrate the probability that a person selects a particular category at a given level of the latent construct. Each curve corresponds to one of the five response options. The figures show that the response alternatives for the respective items are monotonically related to Internet addiction. As one goes from left to right on the *x*-axis, one’s Internet addiction increases.

Figure 2 displays 14 IICs (one for each item). It can be seen that the maximum information is provided by Item 14 followed by Item 6 and Item 10. As for Items 8 and 9, they provide little or no information. The other items are in-between.

The Total (scale) information function (TIF), the sum of item information functions, indicates the precision of the instrument along the latent trait continuum (Figure 3). Here, the term “information” describes reliability or precision of an item or a whole instrument. Reliability and information are linked by formula [92]:

$$reliability=1-\frac{1}{information}$$

It can be seen from Figure 3 that the test provides maximum information for individuals approximately located between *θ* = 0.7 and 1.8. Hence, the reliability estimates in this band is approximately 0.90. As one moves away from this range in either direction, the instrument provides less and less information and consequently becomes less reliable. 

#### 3.3.2. Differential Item Functioning Parameter Estimates

We investigated DIF to evaluate whether the test behaves differently across samples using the R-squared change statistic implemented in the Lordif package. As the output table becomes cumbersome for this number of items (14), we only name the ten (10) that were flagged for DIF. Those are items 2, 4, 5, 6, 7, 8, 9, 12, 13, and 14. This means that these items have either different discrimination parameters (nonuniform DIF) or equal discrimination parameters but different threshold (uniform DIF) value between the three subsamples.

## 4. Discussion

The objectives of this study were to analyze the Arab version of the CIUS by IRT (graded-response) modeling, investigate differential item functioning (DIF) in three samples, as well as further contribute to the ongoing debate related to Internet-use related addictive behaviors using the CIUS items as a proxy.

First, the assumption of unidimensionality of the scale was not supported by the analyses. Second, the presence of local dependency in many items suggest that a multidimensional model might be more appropriate for the fitting of the CIUS scale.

Repeatedly, several studies [57,93] obtain satisfactory fit of a unidimensional construct by letting errors of pairs of items correlate (i.e., Items 1 and 2, 6 and 7, 8 and 9, 10 and 11, 12 and 13). The items concerned by this issue however included some variations across studies (6, 7/12, 13/4, 12/7, 8) [94] or (8, 9, 12). Several studies in Japan [94] and Iran [95] reported three dimensions such as “Difficulties setting priorities” (Items: 1, 2, 3, 4, 12, 13), “Excessive absorption” (Items: 5, 6, 7, 10, 11, 14), and Mood regulation (Items: 8, 9) [94]. The studies (Table 4) which suggested shorter versions of the CIUS took into account such redundancy and tried to maintain the items which are the most appropriately able to catch the latent factor [96,97,98,99]. Such observations and variations across studies may indicate potential differences across samples and may explain some of the between-sample differences reported in the study at hand.

In details, Items 1, 5, and 13 (high discriminative power), then the Items 6, 7, 10, 11, and 14 (very high discriminative power). Among these items, the first one and the eleventh respectively related to impaired control and to continued use despite harm and functional impairment (Table 5); they did not show differences in slope of discrimination parameters across samples. Some of the discriminative items are also commonly retained by different short CIUS scales including Items 1 and 11, as well as the Items 5, 7, and 14. Three of the discriminative items (6, 10, and 13) were, however, not included in any of the short CIUS scales as shown in Table 4. However, we have to consider this observation taking into account the items’ redundancy reported in some of the previous studies showing correlation of variance errors between Item 12 and 13, in addition to between Item 6 and 7, as well as between Items 9 and 10 [61,96].

A high discrimination parameter indicates that the Item has a high ability to give more information on the latent trait [72], allowing for a greater differentiation of people in regard to the latent trait.

Specifically (Table 5), the discriminative items are related to impaired control (Items 1 and 5), increasing priority (Items 6, 7, and 10), continued use despite harm and functional impairment (Item 11), as well as escape (Item 13) and withdrawal (Item 14). The last 2 items were related to symptoms suggested by the DSM-5 but not by ICD-11. Item 5 as well as Item 8 (a non-discriminative item) could be considered as ambiguously categorized between “impaired control” and “continued use despite harm and functional impairment” (i.e., being short of sleep). Items 6 and 7 refers to cognitive aspects of increasing priority which could be also described as “preoccupation”.

As reported in other studies, increasing priority, in its behavioral component, (i.e., Item 10, also referred as loss of interests) [50,100,101], impaired control [50,101], continued use despite harm and functional impairment [101], and withdrawal [100,101] were more endorsed among participants with more severe addictive use of Internet-related behaviors. However, in contradiction with other studies, preoccupation (cognitive component of increasing priority) [50,100,101,102,103] and escape [50,103,104] criteria exhibited good discriminatory power. For instance, these criteria were more frequently endorsed (including among persons with less severe patterns of Internet use) in other studies.

Third, withdrawal is not considered for inclusion in ICD-11 although it is in DSM-5. However, the analysis at hand and a number of other studies [50,100,101,103,105] indicate that withdrawal-related items show a discriminative capability. Yet this symptom is referred to in the CIUS and in other scales as a feeling of irritability or restlessness following cessation of Internet or game use leading to some criticisms about the withdrawal-related construct validity of such items [106]. Tolerance, another symptom suggested by the DSM-5 [41] is not included in the CIUS. The present study is therefore unable to give any information about this controversial symptom [50,100].

The DSM-5 and ICD-11 criteria (Table 5) were related to gaming and not to Internet use. Hence, the Arab translation of the CIUS has to be considered as a proxy measure of the “addictive Internet use” criteria and the findings must be interpreted with caution. In addition, symptoms of Internet addiction were considered as an umbrella construct. For instance, in one network analysis it was reported that symptoms of Internet addiction are often connected with other Internet use-related conditions (i.e., such as gaming disorder) through the same symptoms, suggesting that the Internet is a common vector that mediates specific online behaviors [107]. Assessing specific Internet use (i.e., gaming, cybersex…) would give more precise information in further studies considering the wide range of behaviors connected to Internet use [107].

We also have to consider this study taking into account some strengths and limitations. The major strength is the large sample size and the diversity of the samples. The statistical results as attested by DIF are in line with the geopolitical reality: the Arab population is not a homogeneous entity. Another strength resides in the MML estimation method of IRT modelling: at the model level analysis, the assessment of model fit uses indexes developed specifically for ordinal items. This is not the case in ADF estimation. The study also has limitations. Participants who failed to complete the questionnaire were not analyzed for demographic differences. The samples were not nationally representative and are therefore at risk of self-selection biases [108]. However, the sample used was adequate for the purpose of the study.

## 5. Conclusions

Contrary to earlier ADF estimation findings, unidimensionality of the CIUS scale was not supported by IRT parametric GRM in a large sample of Arab-speaking participants. Other research considering deleting or revising some items are thus necessary to improve the psychometric performance of the scale. For instance, the plethora of item-level detail provided by IRT modelling as well as the correlated error terms can be helpful for a scale revision and guide the determination of the optimal number of factors. Using the test information function, IRT modelling makes it possible to identify where the reliability of the scale is maximal along the latent trait to refine the scale.

The results show that addictive Internet use, as assessed by the CIUS among the Arab-speaking population involves different symptoms, some of them (increasing priority, impaired control, continued use despite harm and functional impairment as well as withdrawal and coping) have greater ability than other CIUS items to discriminate people with higher levels of addictive Internet use. The results must be understood taking into account some of the study limitations. For instance, the CIUS components vary from the DSM-5 or ICD-11 criteria in several ways. Further studies may use longitudinal design, representative samples and combine different assessment tools and clinical interviews for the evaluation of Internet-related addictive behaviors as well as for the assessment of possible concomitant psychiatric disorders or psychological risk factors [34,109]. However, the study may contribute to the debate related to such criteria and add knowledge about Arab-speaking contexts.

## Figures and Tables

**Table 1:** Characteristics of the participants by country measured as mean (SD) for continuous variables and as percentage (%) for categorical variables.

	Algeria *n* = 592	Lebanon, Sample 1 *n* = 155	Lebanon, Sample 2 *n* = 568	Total *n* = 1315
Age				
up to 30	99.9	100	72.7	88.1
31–40	0	0	17.1	7.4
over 40	0	0	4.0	1.7
missing	2.0	0	6.2	2.7
Gender, female	55.1	50.0	73.6	62.4
CIUS total score	33.6 (12.9)	35.9 (8.6)	35.1 (9.0)	34.5 (10.9)

**Table 2:** Item distribution of the CIUS by country.

		Algeria (592)					Lebanon, Sample 1 (155)						Lebanon, Sample 2 (568)					All (1315)					
CIUS	Item Label	1	2	3	4	5	1	2	3	4	5	1		2	3	4	5	1	2	3	4	5	p *
Scoring																							
1	Do you find it difficult to stop using the Internet when you are online?	25.5	22.1	20.3	17.4	14.7	18.7	20.6	34.8	20.0	5.8	14.3		23.6	38.7	7.9	15.5	19.8	22.6	30.0	13.6	14.0	<0.005
2	Do you continue to use the Internet despite your intention to stop?	34.3	18.2	14.9	23.1	9.5	13.5	31.6	32.9	13.5	8.4	10.9		19.9	43.3	15.1	10.7	21.7	20.5	29.3	18.6	9.9	<0.005
3	Do others (e.g., partner, children, parents) say you should use the Internet less?	39.4	19.8	12.3	15.5	13.0	29.0	20.0	21.3	18.7	11.0	25.2		27.6	25.2	8.5	13.6	32.0	23.2	18.9	12.9	13.0	<0.005
4	Do you prefer to use the Internet instead of spending time with others (e.g., partner, children, parents)?	36.8	18.8	17.9	14.0	12.5	11.6	23.9	31.0	23.2	10.3	19.5		28l7	36.6	8.1	7.0	26.4	23.7	27.5	12.5	9.9	<0.005
5	Are you short of sleep because of the Internet?	37.0	18.1	12.3	17.6	15.0	52.9	20.6	11.0	11,6	3.9	41.5		29.2	17.3	4.9	7.0	40.8	23.2	14.3	11.4	10.3	<0.005
6	Do you think about the Internet, even when not online?	56.1	17.2	12.8	8.4	5.4	23.2	35.5	26.5	11.6	3.2	35.2		35.2	20.2	4.6	4.8	43.2	27.1	17.6	7.1	4.9	<0.005
7	Do you look forward to your next Internet session?	41.7	29.4	15.2	10.5	3.2	12.3	27.1	36.1	20.0	4.5	21.1		31.0	30.6	10.2	7.0	29.4	29.8	24.3	11.5	5.0	<0.005
8	Do you think you should use the Internet less often?	32.3	25.5	18.2	14.4	9.5	34.2	29.7	21.3	8.4	6.5	21.5		28.5	30.1	10.7	9.2	27.8	27.3	23.7	12.	9.0	<0.005
9	Have you unsuccessfully tried to spend less time on the Internet?	49.8	19.1	13.0	11.5	6.6	12.3	15.5	21.3	25.8	25.2	11.4		18.7	29.4	17.4	23.1	28.8	18.5	21.1	15.7	15.9	<0.005
10	Do you rush through your (home) work in order to go on the Internet?	46.1	17.7	13.5	14.9	7.8	29.7	26.5	20.6	17.4	5.8	32.7		30.1	24.1	7.6	5.5	38.4	21.1	18.9	12.0	6.5	<0.005
11	Do you neglect your daily obligations (work, school, or family life) because you prefer to go on the Internet?	52.4	17.2	12.2	12.0	6.3	49.0	20.0	14.2	8.4	8.4	40.5		25.5	21.5	6.3	6.2	46.8	21.1	16.4	9.1	6.5	<0.005
12	Do you go on the Internet when you are feeling down?	16.4	16.9	17.1	25.2	24.5	16.8	21.3	31.6	21.9	8.4	23.9		21.3	29.9	14.1	10.7	19.7	19.3	24.3	20.0	16.7	<0.005
13	Do you use the Internet to escape from your sorrows or get relief from negative feelings?	31.6	17.2	16.0	16.6	18.6	21.9	20.6	29.7	19.4	8.4	24.6		23.1	27.3	13.7	11.3	27.5	20.2	22.5	15.7	14.2	<0.005
14	Do you feel restless, frustrated, or irritated when you cannot use the Internet?	45.9	22.5	9.8	12.5	9.3	40.0	22.6	23.9	8.4	5.2	42.8		28.2	18.5	6.2	4.4	43.9	24.9	15.2	9.3	6.7	<0.005

* Not corrected for multiple testing.

**Table 3:** Estimates of discrimination and threshold parameters for the Compulsive Internet Use Scale under the graded response model with the LTM package.

	Discrimination	Threshold			
Item	α_i_	β_1_	β_2_	β_3_	β_4_
1	1.39	−1.33	−0.28	0.96	1.74
2	1.31	−1.27	−0.29	0.93	2.14
3	1.16	−0.80	0.25	1.16	2.03
4	1.21	−1.06	0.03	1.32	2.27
5	1.64	−0.32	0.53	1.15	1.87
6	2.16	−0.21	0.67	1.49	2.16
7	1.74	−0.74	0.33	1.36	2.35
8	0.64	−1.60	0.39	2.24	3.86
9	0.66	−1.42	−0.05	1.38	2.80
10	2.14	−0.35	0.44	1.16	1.96
11	1.85	−0.09	0.64	1.38	2.09
12	1.21	−1.44	−0.44	0.60	1.68
13	1.45	−0.90	−0.07	0.82	1.66
14	2.20	−0.17	0.62	1.26	1.93

**Table 4:** Items with high or very high discriminative power (CIUS-Arab) in comparison with the items of some CIUS short versions.

CIUS	Item Label	CIUS-9 [97]	CIUS-8 [96]	CIUS-5 [98]	CIUS-Arab Items with High or Very High Discriminative Power
1	Do you find it difficult to stop using the Internet when you are online?	X	X	X	X
2	Do you continue to use the Internet despite your intention to stop?				
3	Do others (e.g., partner, children, parents) say you should use the Internet less?	X		X	
4	Do you prefer to use the Internet instead of spending time with others (e.g., partner, children, parents)?	X	X		
5	Are you short of sleep because of the Internet?	X	X	X	X
6	Do you think about the Internet, even when not online?				X
7	Do you look forward to your next Internet session?	X	X		X
8	Do you think you should use the Internet less often?				
9	Have you unsuccessfully tried to spend less time on the Internet?	X	X		
10	Do you rush through your (home) work in order to go on the Internet?				X
11	Do you neglect your daily obligations (work, school, or family life) because you prefer to go on the Internet?	X	X	X	X
12	Do you go on the Internet when you are feeling down?	X	X	X	
13	Do you use the Internet to escape from your sorrows or get relief from negative feelings?				X
14	Do you feel restless, frustrated, or irritated when you cannot use the Internet?	X	X		X

**Table 5:** Suggested classifications of the CIUS-14 items.

CIUS	Item Label	Item Classification by CIUS’ Authors	DSM-5 Concordance *	ICD-11 Concordance *
1	Do you find it difficult to stop using the Internet when you are online?	Loss of Control	Unsuccessful attempt to stop/limit	Impaired control
2	Do you continue to use the Internet despite your intention to stop?	Loss of Control	Unsuccessful attempt to stop/limit	Impaired control
3	Do others (e.g., partner, children, parents) say you should use the Internet less?	Conflict/Negative consequences	Deception	Increasing priority
4	Do you prefer to use the Internet instead of spending time with others (e.g., partner, children, parents)?	Preoccupation regarding Internet use	Loss of interest	Increasing priority
5	Are you short of sleep because of the Internet?	Loss of control	Unsuccessful attempt to stop/limit	Impaired control
6	Do you think about the Internet, even when not online?	Preoccupation regarding Internet use	Preoccupation	Increasing priority
7	Do you look forward to your next Internet session?	Preoccupation regarding Internet use	Preoccupation	Increasing priority
8	Do you think you should use the Internet less often?	Conflict/Problems	Unsuccessful attempt to stop/limit	Impaired control
9	Have you unsuccessfully tried to spend less time on the Internet?	Loss of control	Unsuccessful attempt to stop/limit	Impaired control
10	Do you rush through your (home) work in order to go on the Internet?	Conflict/Problems	Loss of interest	Increasing priority
11	Do you neglect your daily obligations (work, school, or family life) because you prefer to go on the Internet?	Conflict/Problems	Harm/Continue use despite problems	Continued use despite harm and functional impairment
12	Do you go on the Internet when you are feeling down?	Coping/escape	Escape	-
13	Do you use the Internet to escape from your sorrows or get relief from negative feelings?	Coping/escape	Escape	-
14	Do you feel restless, frustrated, or irritated when you cannot use the Internet?	Withdrawal	Withdrawal	-

* Item concordance with DSM-5 and ICD-11 criteria for Internet Gaming and Gaming Disorder (suggested by the authors of the Arab CIUS). In grey: items with high discriminative power; In dark grey: items with the very high discriminative power.

## Data Availability

Data can be made available by the corresponding author upon request.
